# Literature aided determination of data quality and statistical significance threshold for gene expression studies

**DOI:** 10.1186/1471-2164-13-S8-S23

**Published:** 2012-12-17

**Authors:** Lijing Xu, Cheng Cheng, E Olusegun George, Ramin Homayouni

**Affiliations:** 1 Bioinformatics Program, Memphis, TN 38152, USA; 2 Department of Biostatistics, St. Jude Children's Research Hospital, Memphis, TN 38105, USA; 3 Department of Mathematical Sciences, Memphis, TN 38152, USA; 4 Department of Biology, University of Memphis, Memphis, TN 38152, USA

## Abstract

**Background:**

Gene expression data are noisy due to technical and biological variability. Consequently, analysis of gene expression data is complex. Different statistical methods produce distinct sets of genes. In addition, selection of expression p-value (EPv) threshold is somewhat arbitrary. In this study, we aimed to develop novel literature based approaches to integrate functional information in analysis of gene expression data.

**Methods:**

Functional relationships between genes were derived by Latent Semantic Indexing (LSI) of Medline abstracts and used to calculate the function cohesion of gene sets. In this study, literature cohesion was applied in two ways. First, Literature-Based Functional Significance (LBFS) method was developed to calculate a p-value for the cohesion of differentially expressed genes (DEGs) in order to objectively evaluate the overall biological significance of the gene expression experiments. Second, Literature Aided Statistical Significance Threshold (LASST) was developed to determine the appropriate expression p-value threshold for a given experiment.

**Results:**

We tested our methods on three different publicly available datasets. LBFS analysis demonstrated that only two experiments were significantly cohesive. For each experiment, we also compared the LBFS values of DEGs generated by four different statistical methods. We found that some statistical tests produced more functionally cohesive gene sets than others. However, no statistical test was consistently better for all experiments. This reemphasizes that a statistical test must be carefully selected for each expression study. Moreover, LASST analysis demonstrated that the expression p-value thresholds for some experiments were considerably lower (p < 0.02 and 0.01), suggesting that the arbitrary p-values and false discovery rate thresholds that are commonly used in expression studies may not be biologically sound.

**Conclusions:**

We have developed robust and objective literature-based methods to evaluate the biological support for gene expression experiments and to determine the appropriate statistical significance threshold. These methods will assist investigators to more efficiently extract biologically meaningful insights from high throughput gene expression experiments.

## Background

Gene expression data are complex, noisy, and subject to inter- and intra-laboratory variability [1,2]. Moreover, because tens of thousands of measurements are made in a typical experiment, the likelihood of false positives (type I error) is high. One way to address these issues is to increase replicates in the experiments. However this is generally cost prohibitive. Therefore, quality control of gene expression experiments with limited sample size is important for identification of true DEGs. Although the completion of the Microarray Quality Control (MAQC) project provides a framework to assess microarray technologies, others have pointed out that it does not sufficiently address inter- and intra-platform comparability and reproducibility [3-5].

Even with reliable gene expression data, statistical analysis of microarray experiments remains challenging to some degree. Jeffery and coworkers found a large discrepancy between gene lists generated by 10 different feature selection methods, including significance analysis of microarrays (SAM), analysis of variance (ANOVA), Empirical Bayes, and t-statistics [6]. Several studies have focused on finding robust methods for identification of DEGs [7-15]. However, as more methods become available, it is increasingly difficult to determine which method is most appropriate for a given experiment. Hence, it is necessary to objectively compare and evaluate different gene selection methods [6,16-18], which result in different number of DEGs and different false discovery rate (FDR) estimates [19].

FDR is determined by several factors such as proportion of DEGs, gene expression variability, and sample size [20]. Controlling for FDR can be too stringent, resulting in a large number of false negatives [21-23]. Therefore, determination of an appropriate threshold is critical for effectively identifying truly differentially expressed genes, while minimizing both false positives and false negatives. A recent study, using a cross validation approach showed that optimal selection of FDR threshold could provide good performance on model selection and prediction [24]. Although many researchers have made considerable progress in improving FDR estimation and control [25-27], as well as other significance criteria [28-31], the instability resulted from high level of noise in microarray gene expression experiments cannot be completely eliminated. There is therefore a great need to make meaningful statistical significance and FDR thresholds by incorporating biological function.

Recently, Chuchana et al. integrated gene pathway information into microarray data to determine the threshold for identification of DEGs [32]. By comparing a few biological parameters such as total number of networks and common genes among pathways, they determined the statistical threshold by the amount of biological information obtained from the DEGs [32]. This study seems to be the first attempt to objectively determine the threshold of DEGs based on biological function. However, there are several limitations of this study. First, the method relied on Ingenuity pathway analysis which may be biased toward well studied genes and limited by human curation. Second, the threshold selection is iteratively defined. Finally, the approach is manual, which is not realistic for large scale genome-wide applications.

A number of groups have developed computational methods to measure functional similarities among genes using annotation in Gene Ontology and other curated databases [33-38]. For example, Chabalier et al., showed that each gene can be represented as a vector which contains a set of GO terms [34]. Each term was assigned a different weight according to the number of genes annotated by this term and the total number of annotated genes in the collection. Thus, GO-based similarity of gene pairs was calculated using a vector space model. Other studies not only focused on using GO annotations to calculate gene-gene functional similarities but also to determine the functional coherence of a gene set. Recently, Richards et al utilized the topological properties of a GO-based graph to estimate the functional coherence of gene sets [38]. They developed a set of metrics by considering both the enrichment of GO terms and their semantic relationships. This method was shown to be robust in identifying coherent gene sets compared with random sets obtained from microarray datasets.

Previously, we developed a method which utilizes Latent Semantic Indexing (LSI), a variant of the vector space model of information retrieval, to determine the functional relationships between genes from Medline abstracts [39]. This method was shown to be robust and accurate in identifying both explicit and implicit gene relationships using a hand curated set of genes. More recently, we applied this approach to determine the functional cohesion of gene sets using the biomedical literature [40]. We showed that the LSI derived gene set cohesion was consistent across >6000 GO categories. We also showed that this literature based method could be used to compare the cohesion of gene sets obtained from microarray experiments [40]. Subsequently, we applied this method to evaluate various microarray normalization procedures [41]. In the present study, we aimed to develop and test a robust literature-based method for evaluating the overall quality, as determined by functional cohesion, of microarray experiments. In addition, we describe a novel method to use literature derived functional cohesion to determine the threshold for expression p-value and FDR cutoffs in microarray analysis.

## Methods

### Gene-document collection and similarity matrix generation

All titles and abstracts of the Medline citations cross-referenced in the mouse, rat and human Entrez Gene entries as of 2010 were concatenated to construct gene-documents and gene-gene similarity scores were calculated by LSI, as previously described [39,40,42]. Briefly, a term-by-gene matrix was created for mouse and human genes where the entries of the matrix were the log-entropy of terms in the document collection. Then, a truncated singular value decomposition (SVD) of that matrix was performed to create a lower dimension (reduced rank) matrix. Genes were then represented as vectors in the reduced rank matrix and the similarity between genes was calculated by the cosine of the vector angles. Gene-to-gene similarity was calculated using the first 300 factors, which has good performance for large document collections [43].

### Calculation of literature-based functional significance (LBFS)

**Figure 1:**
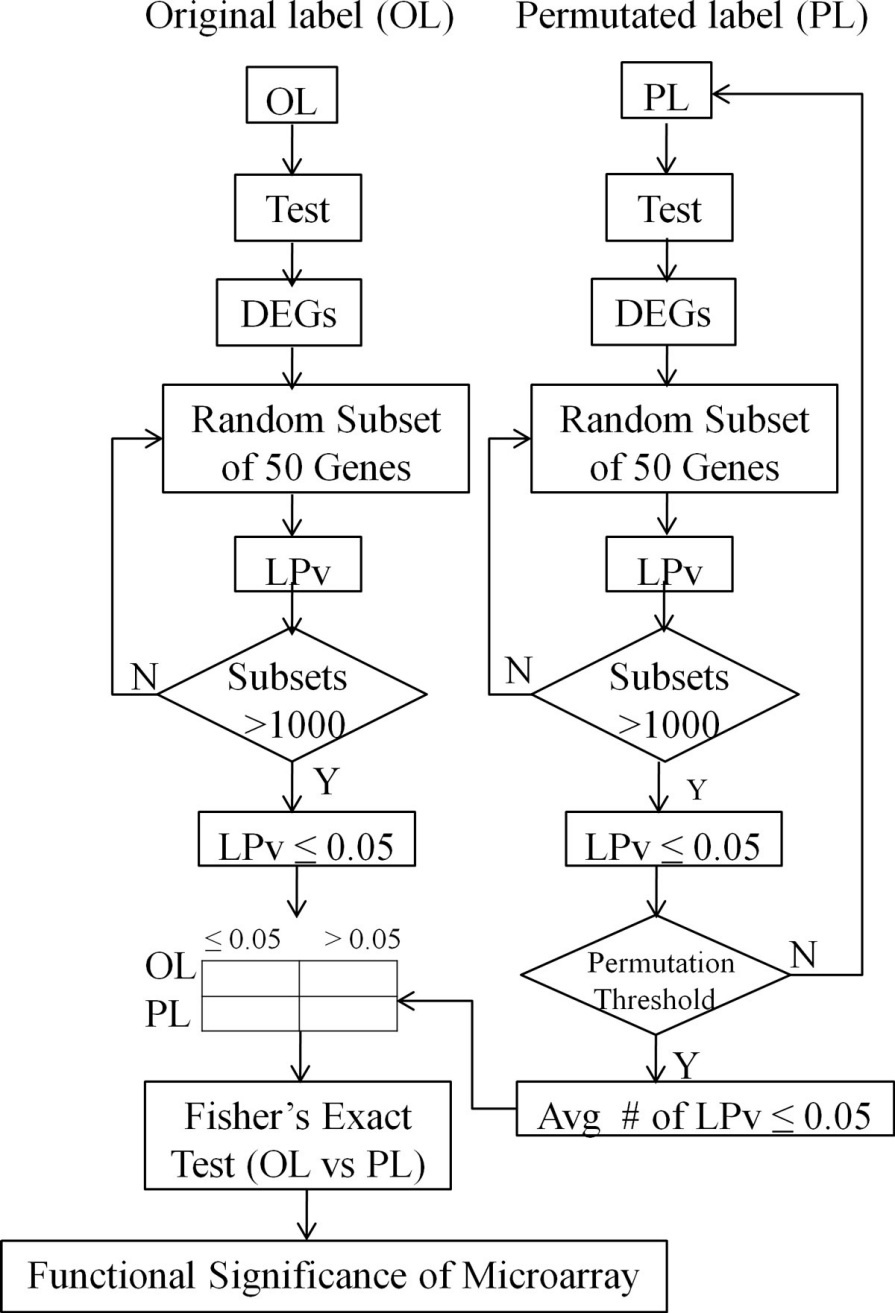
**Overview of the LBFS algorithm**. A statistical test was applied to get differentially expressed genes (DEGs) from the original labeled (OL) and permutated labeled (PL) samples. Subsets of 50 genes were randomly selected 1000 times from each pool of DEGs. Then literature p-values (LPvs) were calculated for each 50 gene-set. A Fisher's Exact test was used to determine if the proportion (called LCI) of subsets with LPv <0.5 in the OL group was significantly different from that obtained from PL group.

This study is an extension of our previous work on gene-set cohesion analysis [40]. Briefly, we showed that LSI derived gene-gene relationships can be used effectively to calculate a literature cohesion p-value (LPv). LPv is derived by using Fisher's exact test to determine if the number of literature relationships above a pre-calculated threshold in a given gene set is significantly different from that which is expected by chance. In many cases, the size of the differentially expressed gene set can be very large. Computationally it is not feasible to calculate one LPv for a very large gene set. Also, it is difficult to compare LPvs if the gene sets are vastly different in size. Therefore, we defined a new metric called *literature cohesion index *(LCI) of randomly sampled subsets of 50 genes from the pool of DEGs. LCI is the fraction of the sampled subsets that have an LPv < 0.05. Then, the overall *literature-based functional significance *(LBFS) of the entire DEG set is determined by a Fisher's exact test comparing the LCI to that expected by chance (i.e., under the complete null hypothesis that no differential expression exists) via a permutation test procedure (Figure 1). In forming the 2-by-2 table, average counts from the multiple permutations are rounded to the nearest integers.

### Literature aided statistical significance threshold (LASST)

**Figure 2:**
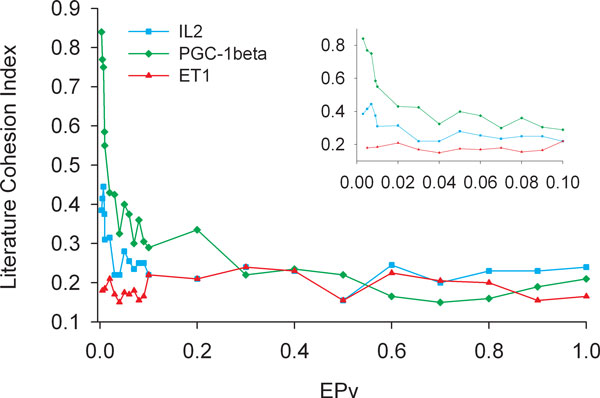
**Relationship between EPV and LCI**. The fraction of gene sets with LPv < 0.05 (y-axis) was plotted at various expression p-value (EPv) thresholds (x-axis) for 3 different datasets. Inset shows magnified view for EPv < 0.10.

Now suppose a differential expression p-value (EPv) is computed for each probe (probeset) by a proper statistical test. A statistical significance threshold (an EPv cutoff) can be determined by considering the relationship between the EPv and the LCI for a given DEG set. First, a grid of EPv cutoffs is specified such as 0.001, 0003, 0.005, 0.01, ⋯, 1, to generate a DEG set at each cutoff value. Next, the LCI is calculated for each DEG set using the sub-sampling procedure as described above. Apart from some random fluctuations, the LCI value is typically a decreasing function of the EPv threshold and assumes an L shape (Figure 2), implying that the LCI partitions the EPv thresholds (and the corresponding DEG sets) into two subpopulations: one with good LCI (the vertical part of the L shape) and one with poor LCI. The EPv threshold at the boundary of the two subpopulations (i.e., at the bend point), can be used as a statistical significance cutoff for selecting DEGs. The bend point can be determined by moving a two-piece linear fit to the L-shaped curve from left to right. The LASST algorithm is as follows:

(1) Specify an increasing sequence of EPv statistical significance thresholds α_1_, ⋯, α_m _and generate DEG sets at these specified significance levels.

(2) For each DEG set generated in (1), estimate the LCI using the sub-sampling procedure described above, to obtain pairs (α_i_, L_i_), i = 1, 2, ⋯, m.

(3) Choose an integer m_0 _(3 by default) and perform two-piece linear fits to the curve as follows: For k = m_0_, m_0_+1, ⋯, m-m_0_, fit a straight line by lease square to the points (α_j_, L_j_), j = 1, 2, ⋯, k (the left piece) to obtain intercept and slope ${\hat{\beta }}_{0k}^{L}$, ${\hat{\beta }}_{1k}^{L}$. Similarly fit a straight line to the points (α_j_, L_j_), j = k+1, 2, ⋯, m (the right piece) to obtain intercept and slope ${\hat{\beta }}_{0k}^{R}$, ${\hat{\beta }}_{1k}^{R}$. Compute $V_{k}=\left({\hat{\beta }}_{0k}^{L}--{\hat{\beta }}_{0k}^{R}\right)+\left({\hat{\beta }}_{1k}^{R}--{\hat{\beta }}_{1k}^{L}\right)$.

(4) Let k* be the first local maxima of V_k _(k == m_0_, m_0_+1, ⋯, m-m_0_), that is, $k^{*}=\text{min}\left\{j:Vj\geq V_{j+1}\right\}$.

(5) Take the k*_th _entry on the α sequence specified in (1) as the EPv significance cutoff.

### Microarray data analysis

To test the performance of our approach, we randomly chose three publicly available microarray datasets from Gene Expression Omnibus (GEO): 1) interleukin-2 responsive (IL2) genes [44]; 2) PGC-1beta related (PGC-1beta) genes [45]; 3) Endothelin-1 responsive (ET1) genes [46]. To be able to compare across these datasets, we focused only on experiments using the Affymetrix Mouse 430-2 platform. All datasets (.cel files) were imported into GeneSpring GX 11 and processed using MAS5 summarization and quantile normalization. Probes with all absent calls were removed from subsequent analysis. As discussed earlier, the content and literature cohesion of a DEG set can largely depend on the statistical test. For this reason, four popular statistical tests including empirical Bayes approach [47], student t-Test, Welch t-Test and Mann-Whitney test were performed to identify DEGs with a statistical significance level 0.05.

## Results

### Comparison of various statistical tests using LBFS

The goal of our study was to develop a literature based method to objectively evaluate the biological significance of differentially expressed genes produced by various statistical methods applied to gene expression experiments. Previously, we developed a method and web-tool called Gene-set Cohesion Analysis Tool (GCAT) which determines the functional cohesion of gene sets using latent semantic analysis of Medline abstracts [40]. However, this method was applicable only to small gene sets and could not be used to compare gene-sets with varying sizes. Here, we have extended this functional cohesion method to determine the biological significance of larger gene sets, which are typically found in microarray studies. To accomplish this, we first calculate the Literature Cohesion Index (LCI, see methods for details) of DEGs produced (Figure 1). Literature based functional significance (LBFS) is then calculated by comparing the LCI of the original labeled experiment and a permuted experiment (Figure 1). Importantly, we found that LBFS values varied greatly between different statistical tests for a given dataset (Table 1). For example, the Empirical Bayes method produced the most functionally significant DEGs for PGC-1beta dataset, but not the other two datasets. In contrast, the Welch t-test generated the most functionally significant DEGs for the IL2 dataset. Both PGC-1beta and IL2 experiments showed significant (p<0.05) LBFS values with multiple statistical tests, whereas none of the tests on ET1 dataset produced DEGs with significant LBFS (Table 1). These results suggest that the PGC-1beta and IL2 experiments likely produced biologically relevant DEGs compared with the ET1 experiments. The lack of biological significance for ET1 DEGs may be due to poor data quality or lack of knowledge in the literature that functionally connects these DEGs. However, the latter may not be the case as the percentage of genes with abstracts was 68-84% for all datasets and statistical tests (Additional file 1).

**Table 1:** Literature based functional significance (LBFS) of gene sets generated by four statistical tests for three different microarray experiments.

		LCI			LBFS	
		
Gene list	PGC-1beta	IL2	ET1	PGC-1beta	IL2	ET1
Welch t-Test	0.34	0.34	0.17	7.08E-06	0.0004	0.45
Mann-Whitney	0.2	0.2	0.13	0.118	0.0075	1
Student t-Test	0.38	0.38	0.1	1.24E-07	0.071	1
Empirical Bayes	0.4	0.19	0.05	1.36E-08	0.11	1

For comparison the Literature Cohesion Index (LCI) which is used to calculate LBFS is displayed for each experiment.

### Determination of EPv threshold using LASST

In the above analysis, DEGs were selected using an arbitrary statistical threshold of p<0.05, as is the case for many published expression studies. However, in reality, there is no biological reason why this threshold is selected for experiments. Once the appropriate statistical test was chosen by application of LBFS above, we tested if literature cohesion could be applied to determine the EPv cutoff. We developed another method called Literature Aided Statistical Significance Threshold (LASST) which determines the EPv by a two-piece linear fit of the LCI curves as a function of EPv as described in Methods. LASST was applied to p-values produced by Empirical Bayes for PGC-1beta experiment and Welch t-test for the IL2 and ET1 experiments. DEGs were produced at each point on a grid of unequally-spaced statistical significance levels (α = 0.001, 0.003, 0.005,⋯). In computing the LCI, the LPv level was set to 0.05, and the size of the gene subsets from the DEG pool was set to 50 in the sub-sampling procedure as described in Methods. The LCI of a DEG set was plotted against various α levels of the EPv (Figure 2). Interestingly, application of LASST determined an EPv significance threshold of 0.01 (corresponding LCI 0.55) for PGC-1beta dataset and 0.02 (LCI 0.315) for IL2 dataset. None of the DEG sets from the ET1 experiment had appreciable LCI, which remained consistently low across the α levels (Figure 2). Thus, an EPv threshold could not be determined using the LCI approach for ET1 dataset. These results are consistent with what we observed above (Table 1).

**Figure 3:**
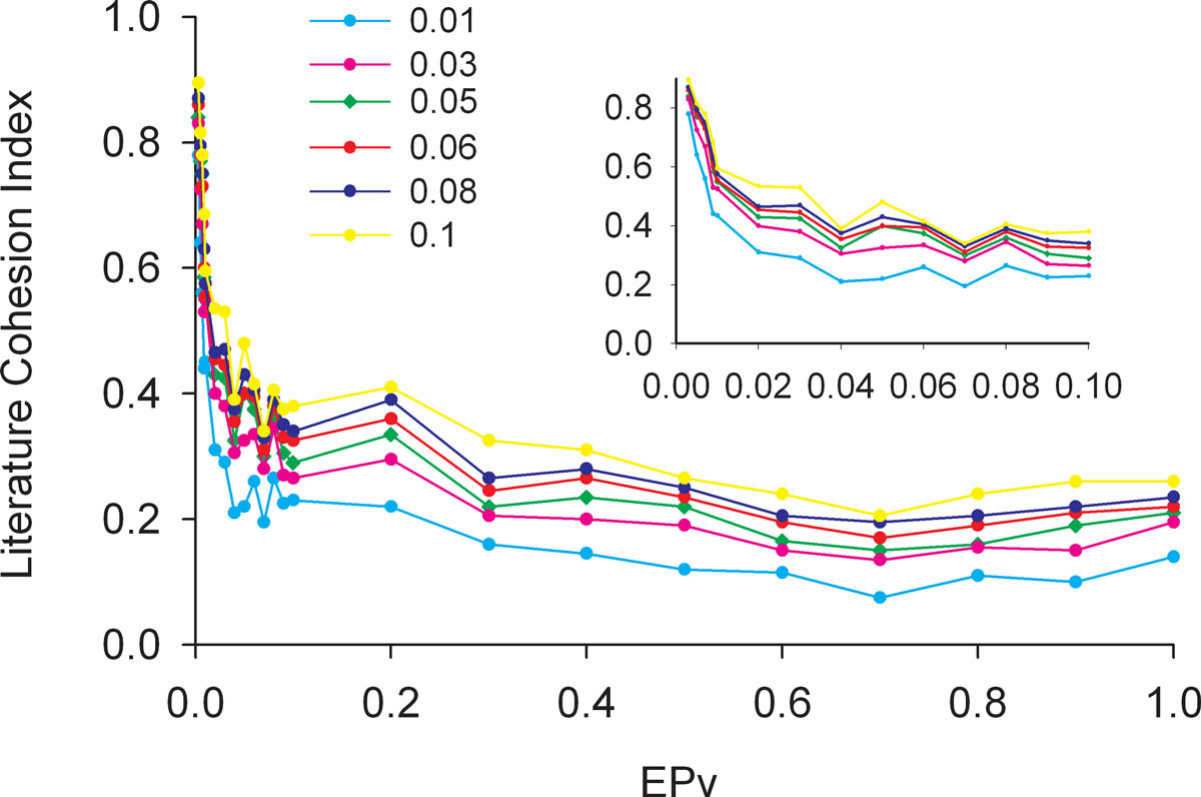
**Relationship between EPV and LCI at various thresholds**. The LCI at various LPv thresholds ranging from 0.01 to 0.1 (y-axis) was plotted against various EPv thresholds (x-axis) for PGC-1beta dataset. Inset shows magnified view for EPv < 0.10. The shapes of the curves are similar at various LPv thresholds.

While computing LCIs in the above analysis, the LPv threshold was set at 0.05. We wondered if different LPv thresholds would affect LASST results. Therefore, we calculated LCI at different LPv thresholds such as 0.01, 0.03, 0.05, 0.06, 0.08 and 0.1. We found that the shape of the LCI curves were similar with respect to EPv values (Figure 3), indicating that LASST is not sensitive to different reasonably conservative LPv thresholds.

We next compared the LASST results with several popular multiple hypothesis testing correction procedures along with the unadjusted p-value threshold of 0.05 in a student t-test (Table 2). For the IL2 experiment, Storey's q-value method at 0.1 identified the highest number of DEGs. In stark contrast, only 1 gene was selected by any of the four FDR correction methods for the PGC-1beta experiment and 0 genes were selected for the ET1 experiment. Importantly, application of LASST selected 3485 genes at a p-value threshold of 0.02 (corresponding to FDR 0.032) and 1175 genes at a p-value threshold of 0.01 (corresponding to FDR 0.074) for IL2 and PGC-1beta experiments, respectively. These results suggest that perhaps more biologically relevant DEGs can be selected with lower FDR values.

**Table 2:** Number of significant genes identified by student t-test after correction for multiple hypotheses testing

	# of tests	# of genes with p <0.05	Storey pFDR q<0.1	BH FDR <0.1	Bonferroni FWER <0.1	Westfall Young Permutation
IL2	20558	5001	5955	3827	32	95
PGC-1beta	17633	2618	1	1	1	1
ET1	20477	1559	0	0	0	0

## Discussion

Although microarray technology has become common and affordable, analysis and interpretation of microarray data remains challenging. Experimental design and quality of the data can severely affect the results and conclusions drawn from a microarray experiment. Using our approach, we found that some datasets (e.g., PGC-1beta) produced more functionally cohesive gene sets than others (e.g., ET1). There can be many biological or technological reasons for the lack of cohesion in any microarray dataset. For instance, it is possible that the experimental perturbation (or signaling pathway) simply did not alter mRNA expression levels in that system as hypothesized. It is also possible that the data are noisy due to technical or biological variations, which result in false differential expression. Although our method will not identify the causes of this variation, it can help in assessment of the overall quality of the experiment and provide feedback to the investigators in order to adjust the experimental procedures. For example, after observing a low LBFS value, the investigator may choose to remove outlier samples or add more replicates into the study design.

It is important to note that a low cohesion value could be due to a lack of information in the biomedical literature. In other words, it is possible that the microarray experiment has uncovered new gene associations which have not been previously reported in the literature. This issue would affect any method that relies on human curated databases or natural language processing of biomedical literature. However, our LSI method presents a unique advantage over other approaches because it extracts both explicit and implicit gene associations, based on weighted term usage patterns in the literature. Consequently, gene associations are ranked based on their conceptual relationships and not specific interactions documented in the literature. Thus, we posit that LSI is particularly suited for analysis of discovery oriented genomic studies which are geared toward identifying new gene associations. Further work is necessary to be able to determine exactly how (whether explicitly or implicitly) a subset of functionally cohesive genes are related to one another in the LSI model.

A major challenge in microarray analysis involves selection of the appropriate statistical tests, which have different assumptions about the data distribution and result in different DEG sets. For instance, parametric methods are based on the assumption that the observations adhere to a normal distribution. The assumption of normality is rarely satisfied in microarray data even after normalization. Nonparametric methods are distribution free and do not make any assumptions of the population from which the samples are drawn. However, nonparametric tests lack statistical power with small samples, which is often the case in microarray studies. In this study, we found that although Mann-Whitney nonparametric test identified the largest number of DEGs for PGC-1beta experiment, the DEGs were not functionally significant (Table 1). Also, we found that some tests were selectively better for some experiments. For example, the Empirical Bayes method produced the best results for the PGC-1beta experiment, while the Welch t-test produced the best results for the IL2 experiment. Taken together, we demonstrate that our method allows an objective and literature based method to evaluate the appropriateness of different statistical tests for a given experiment.

Several groups have developed methods to assess functional cohesion or refine feature selection by incorporating biological information from either the primary literature or curated databases [38,48-50]. To our knowledge, a literature based approach to evaluate the overall quality of microarray experiments has not been reported. Although we did not extensively compare our approach with these methods, we performed a preliminary comparison with a well known Gene Set Enrichment Analysis (GSEA) method [49]. GSEA calculates the enrichment p-value for biological pathways in curated databases for a given set of DEGs. Presumably, if a microarray experiment is biologically significant, then higher number of relevant pathways should be enriched. Indeed, we found that GSEA identified 410, 309 and 283 enriched pathway gene sets with FDR <0.25 for PGC-1beta, IL2 and ET1experiments, respectively. These results correlated well with our LBFS findings which showed that DEGs obtained from PGC-1beta and IL2 were more functionally significant than ET1. However, GSEA identified a substantial number of enriched pathways for ET1. One issue is that GSEA only focuses on gene subsets and not the entire DEG list. Thus, it does not evaluate the overall cohesion or functional significance of the DEG list. In addition, since GSEA relies on human curated databases such as GO and KEGG, it is susceptible to curation biases, which favor well-known genes and pathways and contain limited information on other genes.

Assuming that microarray experiment is of high quality and an appropriate statistical test has been selected for a microarray experiment, selection of the expression p-value cutoff still remains arbitrary for nearly all published studies. In our work, we found a positive correlation between literature cohesion index and EPv (Figure 2). Based on the distribution of LCI with respect to EPv, we devised a method (called LASST) which empirically determined the EPv cutoff value. Not surprisingly, we found that different EPv cutoffs should be used for the different microarray experiments that we examined. Indeed, we found that application of LASST resulted in a smaller p-value threshold and substantially smaller number of DEGs for both IL1 and PGC-1beta experiments. Therefore, LASST enables researchers to narrow their gene lists and focus on biologically important genes for further experimentation.

Finally, another major challenge for microarray analysis is the propensity for high false discovery rate (FDR) caused by multiple hypothesis testing. Correction of multiple hypothesis testing including family wise error rate (FWER) are often too stringent which may lead to a large number of false negatives. As with EPv cutoff concerns above, setting the FDR threshold at levels 0.01, 0.05, or 0.1 does not have any biological meaning [29]. For instance, no false positive error correction method produced adequate DEGs for PCG-1beta and ET1 experiments. However, our analysis showed that PGC-1beta dataset was biologically very cohesive (Table 1). This suggests that applying FDR correction to this dataset would produce a very large number of false negatives. Another important finding of our study is that the false positive error correction procedures appear to be sensitive to DEG size. For instance, using student t-test IL2 dataset consisted of 5001 DEGs with a p-value <0.05, whereas the Storey FDR method produced 5955 at q<0.1. However, our literature based analysis revealed that the IL2 dataset produced less biologically cohesive DEGs than the PGC-1beta dataset, which showed only 1 gene with q<0.1. In the future, it will be important to expand these preliminary observations to a larger of set of microarray experiments and to determine the precise relationships between false positive correction methods and biological significance.

## Conclusions

In this study, we developed a robust methodology to evaluate the overall quality of microarray experiments, to compare the appropriateness of different statistical methods, and to determine the expression p-value thresholds using functional information in the biomedical literature. Using our approach, we showed that the quality, as measured by the biological cohesion of DEGs, can vary greatly between microarray experiments. In addition, we demonstrate that the choice of statistical test should be carefully considered because different tests produce different DEGs with varying degrees of biological significance. Importantly, we also demonstrated that procedures that control false positive rates are often too conservative and favor larger DEG sets without considering biological significance. The methods developed herein can better facilitate analysis and interpretation of microarray experiments. Moreover, these methods provide a biological metric to filter the vast amount of publicly available microarray experiments for subsequent meta-analysis and systems biology research.

## Abbreviations

ANOVA: analysis of variance; DEGs: differentially expressed genes; EPv: expression p-value; ET1: Endothelin-1 responsive; FDR: False Discovery Rate; GCA: gene-set cohesion analysis; GCAT: Gene-set Cohesion Analysis Tool; GEO: Gene Expression Omnibus; IL2: interleukin-2 responsive; LASST: Literature aided statistical significance thresholds; LBFS: literature-based functional significance; LCI: literature cohesion index; LPv: literature cohesion p-value; LSI: Latent Semantic Indexing; MAQC: Microarray Quality Control; PGC-1beta: PGC-1beta related; SAM: significance analysis of microarrays; SVD: singular value decomposition;

## Competing interests

The authors declare that they have no competing interests.

## Authors' contributions

L. Xu developed the algorithm, carried out the data analyses, performed all of the evaluation and wrote the manuscript. C. Cheng developed the literature aided statistical significance thresholds method and wrote part of the manuscript. E.O. George provided statistical supervision of the study. R. Homayouni conceived, co-developed the methods, supervised the study and wrote the manuscript.

## Supplementary Material

Additional file 1 **Number of DE genes (with 0.05 EPv) and percentage of having abstracts that generated from different tests for PGC-1beta, IL2 and ET1 datasets**. Click here for file
