# Supplementary material for: Literature aided determination of data quality and statistical significance threshold for gene expression studies
Source: BMC Genomics. 2012 Dec 17;13(Suppl 8):S23. doi: 10.1186/1471-2164-13-S8-S23 (PMC3535704; doi:10.1186/1471-2164-13-S8-S23)
Supplement: Additional file 1 — Number of DE genes (with 0.05 EPv) and percentage of having abstracts that generated from different tests for PGC-1beta, IL2 and ET1 datasets. [file 1471-2164-13-S8-S23-S1.pdf]

Additional file 1. Number of DE genes (with 0.05 EPv) and percentage of having abstracts that generated from different tests for PGC-1beta, IL2 and ET1 datasets.

	# of DE Genes			# of DE Genes with Abstracts			Ratio of DE Genes with Abstracts		
Gene list	PGC-1beta	IL2	ET1	PGC-1beta	IL2	ET1	PGC-1beta	IL2	ET1
Welch t-Test	2045	4761	1541	1634	3847	1293	0.80	0.81	0.84
Mann-Whitney	3877	4498	2905	3152	3625	2367	0.81	0.81	0.81
Student t-Test	2618	5001	1559	2075	3951	1291	0.79	0.79	0.83
Empirical Bayes	2933	5133	304	2381	4104	207	0.81	0.80	0.68
